# A Mini-Review of the Main Effects of Essential Oils from *Citrus aurantifolia*, *Ocimum basilicum*, and *Allium sativum* as Safe Antimicrobial Activity in Poultry

**DOI:** 10.3390/ani14030382

**Published:** 2024-01-25

**Authors:** Gabriel da Silva Oliveira, Concepta McManus, Heloisa Alves de Figueiredo Sousa, Pedro Henrique Gomes de Sá Santos, Vinícius Machado dos Santos

**Affiliations:** 1 Faculty of Agronomy and Veterinary Medicine, University of Brasília, Brasília 70910-900, Brazil; gabriels.unb@gmail.com (G.d.S.O.);; 2 Laboratory of Microbiology and Food, Federal Institute of Brasília—Campus Planaltina, Brasília 73380-900, Brazil; 3 AVF/SA, Brasília 71503-501, Brazil; 4 Laboratory of Poultry Science, Federal Institute of Brasília—Campus Planaltina, Brasília 73380-900, Brazil

**Keywords:** antimicrobials, essential oils, health protection, microbiological safety, microorganisms, natural product

## Abstract

**Simple Summary:**

Increasing the use of natural antimicrobials and introducing them into the poultry production cycle is desirable. Essential oils are promoted as one of the main alternatives to intensive conventional antibiotic therapy in poultry farming worldwide. Studies show that when applied in specific forms and concentrations, several essential oils or their components have demonstrated the ability to protect both humans and poultry from serious diseases, including those associated with microorganisms. For example, in theory and practice, salmonellosis is widely recognised as a significant concern for human and avian health. In this context, acquiring knowledge about essential oils that can potentially prevent or combat microbial outbreaks in poultry farming, leading to serious infectious complications for avian and human health, is crucial. This review aimed to compile information on the application of *Citrus aurantifolia* (CAEO), *Ocimum basilicum* (OBEO), and *Allium sativum* (ASEO) essential oils as antimicrobials in poultry farming.

**Abstract:**

Poultry production is accompanied by the use of antimicrobial agents because no production step is free of microorganisms. In the absence of antimicrobial treatments with synthetic drugs, essential oils are among the most cited natural alternatives used to prevent and treat microbial contamination in poultry. Although there are several studies on the antimicrobial properties of essential oils, there is still no review that simultaneously compiles information on the leading antimicrobial role of essential oils from *Citrus aurantifolia* (CAEO), *Ocimum basilicum* (OBEO), and *Allium sativum* (ASEO) in poultry. Awareness of the antimicrobial role of these substances opens the door to encouraging their use in natural antimicrobial protocols and discouraging harmful synthetics in poultry. This review aimed to compile information on applying CAEO, OBEO, and ASEO as antimicrobials in poultry farming. The available literature suggests that these essential oils can proportionately align with the poultry industry’s demands for microbiologically safe food products.

## 1. Introduction

In contaminated poultry environments, which include contact surfaces, soil, air, and water, among others, the chances of contaminating poultry and their products are high. This scenario can trigger a sequence of interconnected events. Poultry can develop severe disease symptoms or die when they are exposed to contamination. For example, poultry contaminated with *Escherichia coli* may present an infectious condition associated with movement restrictions and nutrient deprivation [1], leading to death. However, regardless of the severity of the symptoms—whether intense, mild or even absent, poultry can become asymptomatic carriers, with contamination maintained in a latent state until the final product (meat or egg) is obtained. This process can compromise the productivity and viability of commercialisation. Thus, the impacts of microorganisms such as *Salmonella* spp., *Campylobacter* spp., *Staphylococcus aureus*, *Escherichia coli*, *Aspergillus* spp., and *Fusarium* spp. on broilers or laying hens cannot go unnoticed, as these poultry animals sustain the needs of populations internationally.

Sanitisation and other protocols that use effective and safe antimicrobials interrupt the different cycles of contamination that compromise the productivity and quality of poultry food products, ensuring food availability and keeping consumers safe from contaminated food. These cycles start from the contaminated environment and then reach humans, as shown below:

- Environment → poultry → table eggs → humans.
- Environment → poultry → hatching eggs → embryos → poultry → meat → humans.
- Environment → poultry → meat → humans.
- Environment → hatching eggs → embryos → poultry → meat → humans.
- Environment → hatching eggs → embryos → poultry → table eggs → humans.
- Environment → table eggs → humans.
- Environment → meat → humans.

Essential oils comprise blends of aromatic compounds extracted from different parts of plants, such as fruits, roots, rhizomes, leaves, flowers, bark, buds, twigs, wood, and seeds, known for their distinctive fragrances [2]. Certain essential oils have been investigated for microbiological control in poultry [3,4,5,6]. Among these, the essential oils of *Citrus aurantifolia* (CAEO), *Ocimum basilicum* (OBEO), and *Allium sativum* (ASEO) stand out [7,8,9], mainly for their natural and antimicrobial characteristics, which shape their sustainable properties for probable productive gains and benefits to human and environmental health. Therefore, the strategic and conscious administration of these essential oils in the poultry sector involves innovative protocols for solving microbiological problems, favouring sustainable practices. This review compiles information on applying CAEO, OBEO, and ASEO as antimicrobials in poultry farming. The following issues were addressed: which bacteria and fungi can be inhibited using essential oils; and the primary uses of essential oil antimicrobial properties for different purposes in poultry.

## 2. The Choice of Essential Oils and Methodology for Collecting Studies in the Available Database

Due to the usage requirements and selection criteria, there was no single and definitive reason for choosing these CAEO, OBEO, and ASEO essential oils to study in poultry production. However, at least eight reasons were decisive in selecting essential oils:

→ Authors’ research materials.
→ Potential innovative applications in poultry production stages.
→ Specific chemical composition.
→ Meet the specific requirements of the study objective.
→ Functions that serve the industrial field.
→ Commercial availability and practical application.
→ Advantageous competitive efficiency compared with different conventional products.
→ Economic viability, potential benefit, and minimal risks.

All scientific documents cited in this study were found on Google Scholar: https://scholar.google.com/ (accessed on 17 October 2023).

The keywords or sentences used to search scientific documents were the following:

→ *Citrus aurantifolia* (CA).
→ CAEO.
→ In vitro antimicrobial (or antibacterial/antifungal) effects of CAEO.
→ Toxicity (or safety) of CAEO.
→ CAEO and poultry.
→ Antimicrobial effect of CAEO in poultry.
→ *Ocimum basilicum* (OB).
→ OBEO.
→ In vitro antimicrobial (or antibacterial/antifungal) effect of OBEO.
→ Toxicity (or safety) of OBEO.
→ OBEO and poultry.
→ Antimicrobial effects of OBEO in poultry.
→ *Allium sativum* (AS).
→ ASEO.
→ In vitro antimicrobial (or antibacterial/antifungal) effects of ASEO.
→ Toxicity (or safety) of ASEO.
→ ASEO and poultry.
→ Antimicrobial effects of ASEO in poultry.

The criteria used to review the documents found in full were as follows:

→ Research, review and conference papers, book chapters and monographs.
→ Written in English or Portuguese.
→ Published in any year.
→ Title, abstract, and keywords that meet the idea proposed for each topic of this study.

## 3. *Citrus aurantifolia* (CA), *Ocimum basilicum* (OB), and *Allium sativum* (AS) Plants and Their Essential Oils

Plants present an incredible bank of functional compounds. Each plant has its own individual characteristics, regardless of the species or variety. They are easily influenced by a considerable list of environmental factors, including density, photoperiod, and temperature [10,11]. Three of the most popular plants are CA, OB, and AS. These plants provide essential oils highly valued by the scientific community, industry, and consumers. 

CA is a perennial fruit tree with an average height of 5 m belonging to the Rutaceae family [12]. Its leaves are greenish with an elliptical to oblong-ovate shape and can reach 9 cm in length [12,13]. It produces a greenish-yellow sour fruit with a smooth surface, approximately 4 cm long and 4 cm in diameter, weighing 41 g [13,14]. The fruits are harvested in substantial quantities 4 to 8 years after planting the seedlings [13]. The CA tree produces fruit annually and adapts well to different climates and soil conditions, requiring low input costs and good market demand [15]. CAEO is an aromatic liquid mainly extracted from the peels and leaves of CA through various extraction protocols, which include maceration, solvent (Soxhlet), hydrodistillation, and steam distillation [16,17,18,19,20]. This oil can yield 18.02% when extracted by Soxhlet [21], a density of 0.86 g/cm^3^, and a refractive index of 1.48 at 20 °C [19]. Its colouration can range from colourless to greenish-yellow or yellowish-green [19]. The main component of CAEO is limonene, representing up to 98.3% of the essential oil [22]. In addition to limonene, at least 17 other compounds can make up the complex chemical arrangement of CAEO [16]. Linalool, β-Pinene, γ-Terpinene, citronellal, and citronellol are other compounds that comprise a significant proportion of this essential oil [16,23]. 

OB is an annual aromatic herb with heights and weights ranging from approximately 26 to 59 cm and 76 to 203 g, respectively, and belonging to the Lamiaceae family [24,25,26]. Its leaves are oval, pointed, and opposite, and in most varieties, they are green, reaching approximately 11 cm in length and 8 cm in width [24,26]. The flowers are predominantly white but can be other colours, such as pink and purple. Each herb can have up to approximately 50 inflorescences, reaching about 19 cm in length [24]. OB can be grown without problems in various environmental conditions [25]. OBEO is a liquid storehouse of active compounds that can be extracted from OB’s leaves, stems, and flowers via hydrodistillation and steam distillation [27,28,29]. This oil can have a yield of 2.26% when extracted by hydrodistillation [30], a density of 1.20 g/cm^3^, a viscosity of 14.37 g^−1^·cm·s, and a refractive index of 1.64 at 20 °C [31]. Its colouration is usually yellowish [30]. OBEO can be chemically formed by just 2 compounds or up to 23 [32], depending on the conditions in which the plant from which it originated was cultivated [32]. Estragole (60.98%) and Linalool (41.2%) constitute the significant chemical portion of OBEO [28,33]. Other compounds, such as methyl chavicol and methyl-eugenol, can also be detected in significant concentrations [27,28]. 

AS is a bulbous perennial herb belonging to the Lilliceae family [34] capable of reaching 117 cm in height [35]. Each bulb can contain up to 42 white cloves and can weigh up to 257 g [35]. A plant can produce 13 leaves [35] characterised as greenish, elongated, and flattened from the bulb to pointed at the tip [36]. AS grows well in heavy clay soils enriched with humus and water [36]. ASEO is a yellowish concentrate of natural chemical compounds [37] that can be extracted from the clove, aerial part, and bulb of AS through steam distillation [38,39,40]. This oil can yield 22.5% when extracted by Soxhlet [41], a density of 1.03 g/cm^3^, and a refractive index of 1.47 at 20 °C [37]. Diallyl disulfide or allicin commonly constitute a major part of the chemical composition of ASEO [39,40]. Other identified compounds that contributed significantly to the total content of this oil are 2-methoxy-4-vinylphenol, decene, allyl propyl disulfide, allyl methyl trisulfide, di-2-propenyl-trisulfide, diallyl tetrasulfide, and siloxane [38,39,40]. 

## 4. The Antimicrobial Efficacy of Essential Oils from *Citrus aurantifolia* (CAEO), *Ocimum basilicum* (OBEO), and *Allium sativum* (ASEO), and Their Applications in Poultry

**Figure 1:**
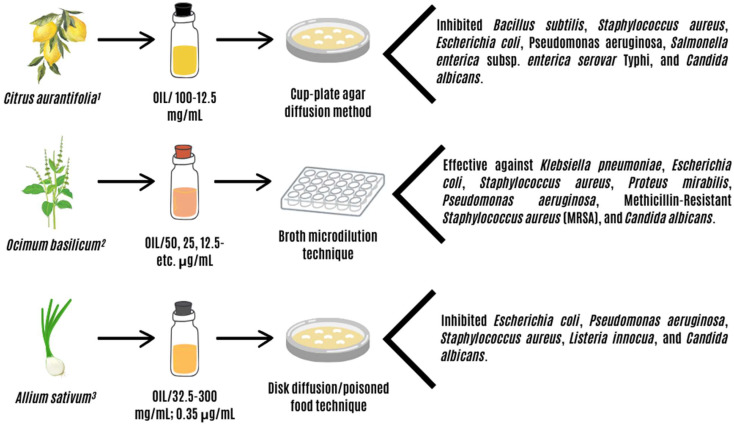
Findings on the antimicrobial efficiency of essential oils from *Citrus aurantifolia* (CAEO), *Ocimum basilicum* (OBEO), and *Allium sativum* (ASEO). Sources: ^1^ [42]; ^2^ [43]; ^3^ [44,45].

CAEO, OBEO, and ASEO open up a network of possibilities to be applied as antibacterial and antifungal agents thanks to in vitro results that support their effectiveness (Figure 1).

The mechanisms of action against microorganisms that justify the antimicrobial characteristics of these essential oils have already been studied and described [46,47,48]. Musdja et al. [46] reported that CAEO causes cumulative bacterial cell destruction events. It starts by damaging the membrane, followed by the leakage of proteins, nucleic acids, and K^+^ and Ca^2+^ ions. Dysfunctions induced by this essential oil yield bacterial cells of irregular sizes with debris [49]. As the main compound of CAEO, limonene has demonstrated its antibacterial potential due to, among other things, enzyme inhibition and regulation, the disruption of translation in protein synthesis, and the inhibition of cell wall synthesis [50]. Similar events were also found when investigating the mechanism of action of limonene against fungi [51]. This demonstrates this compound’s significant contribution to the antimicrobial potential of CAEO. When in contact with bacteria, OBEO can compromise cell structure and integrity, resulting in the leakage of macromolecular substances and intracellular ionic electrolytes [48]. Although this mechanism of action against bacterial survival is not linked to just one compound, the significant participation of linalool in this antimicrobial process was emphasised by Li et al. [48]. The antifungal potential of OBEO may be related to the inhibition of yeast transformation into hyphae [52]. Bacteria exposed to ASEO show an increase in the permeability of their cell membrane, and the entire environment responsible for their survival and metabolism is impaired, making their survival impossible [47]. Fungi exposed to ASEO are killed because their cell membranes and organelles (e.g., mitochondria) are damaged [45]. Allicin is one of the representative points of origin for the antimicrobial capacity of AS essential oil [53]. In situations where allicin is present in low concentrations in AS oil, other compounds, such as Diallyl disulfide, can effectively reinforce the antimicrobial function of ASEO [54].

Natural antimicrobials with different colours, smells, compositions, biological effects, toxicity levels, and countless applications can be developed from trees, shrubs, herbs, and other plant varieties worldwide. Some of these antimicrobials are CAEO, OBEO, and ASEO, which have been suggested for use in poultry production to ensure that poultry food products (meat and eggs) have minimal microbial loads. This should not incur sudden changes in appearance, ensure a long shelf life, and ensure that the food can be safely consumed for an extended period. The ability of these three essential oils to provide microbial control in eggs and poultry meat, alone or in combination with other substances or formulations, has been tested; a summary of the findings is reported in Table 1.

The antimicrobial effects of essential oils have also been used in dietary interventions for poultry from the initial stage of life after hatching. This approach aims to balance the diet beyond the conventional standard, offering nutritional support that beneficially promotes the development, survival, productivity, and, mainly, the health of poultry. Elnaggar and El-Tahawy [58] observed that providing diets containing OBEO (0.5 g and 1 g of oil/kg of feed) resulted in significant improvements in the growth, feed conversion, economic efficiency, production index, immune response, carcass characteristics, and general health of broiler chickens when compared with the non-supplemented control group. Additionally, there were reductions in total bacterial counts, *Salmonella* spp., *Escherichia coli*, and *Proteus* spp. in the digestive system of broiler chickens compared with the control group. Similarly, one study demonstrated that the strategic introduction of 0.05% OBEO oil into the diet of broiler chickens increased the concentration of beneficial bacteria (e.g., *Lactobacillus*) and reduced harmful bacteria (e.g., *Escherichia coli*) in the intestine and cecum, which was reflected in an improved feed conversion ratio [59]. Abd El-Latif et al. [60] also observed that the provision of poultry feed plus 100 mg of ASEO/kg of feed improved the performance of broiler chickens and stimulated innate immunity. In microbiological terms, the intestinal health of broiler chickens benefited when they consumed a diet with nanoencapsulated ASEO in the same concentrations [61]. As indicated by Elbaz et al. [62], dietary supplementation with ASEO (200 mg of oil/kg diet) improved growth performance and enhanced carcass characteristics, nutrient digestion, blood lipid metabolism, and intestinal microbiota. We found no studies that evaluated the use of CAEO in poultry nutrition.

## 5. Conclusions and Future Perspectives

Existing findings reinforce the concept that essential oils can proportionately align with the poultry industry’s demands for microbiologically safe food products. However, antimicrobial measures should not be limited exclusively to final poultry products. Such measures must be used throughout the production chain. For example, healthy chickens for consumption are likely to come from properly handled healthy embryos/chicks [63]. Proper management needs to involve antimicrobial protocols as a form of prevention. These protocols must be implemented from the beginning of the production process, with sanitisation processes of hatching eggs. After hatching, providing a diet that includes natural antimicrobials is essential, maintaining this approach until the final product is consumed. Based on knowledge of the safety and antimicrobial efficacy of CAEO, OBEO, and ASEO, it is hypothesised that these are potent alternatives for safe microbiological control in all sectors of the poultry chain. During the poultry breeding phase, microbiological control is strongly associated with the preservation of health. Poultry professionals and researchers need to fill knowledge gaps with investigations aimed at producing state-of-the-art antimicrobials based on CAEO, OBEO, and ASEO that can promote healthy poultry farming, explicitly targeting the pathogenic microbiota of poultry products and ensure the integrity of products from the poultry sector and human health. Advancing improvements in poultry management and understanding the need for green antimicrobials in poultry farming is a big step towards achieving these goals.

## Figures and Tables

**Table 1:** Antimicrobial effects of essential oils from *Citrus aurantifolia* (CAEO), *Ocimum basilicum* (OBEO), and *Allium sativum* (ASEO) in poultry products.

Essential Oil	Concentration	Application Method	Poultry Product	Food Image	Findings	Reference
CAEO	200 and 400 mg/mL	Spraying	Chicken meat	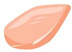	Reduced counts of *Escherichia coli* (−7.90 log), *Salmonella enterica* subsp. *enterica serovar* Typhi (−5.30 log) and *Salmonella enterica* subsp. *enterica serovar* Typhimurium (−3.10 log).	[8]
CAEO	1%	Immersion	Egg	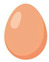	Reduced the total number of aerobic mesophilic bacteria in the shell (−0.92 log) and content (−1.04 log).	[55]
OBEO	0.20%	Vacuum packaging	Chicken thighs	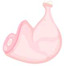	Reduced the population of anaerobic bacteria (−1.34 log) and lactic acid bacteria (−0.62 log) and bacteria from the Enterobacteriaceae family (−1.55 log).	[56]
OBEO	2.5 and 5.0 mg/mL	Immersion	Chicken meat	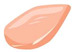	Reduced the number of *Salmonella enterica* subsp. *enterica serovar* Enteritidis (−1.15–2.46 log).	[7]
OBEO	300 mg/mL	Immersion	Egg	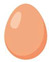	Reduced the number of aerobic mesophilic bacteria, Enterobacteriaceae, moulds, and yeasts in the shell (−1.71 log, −1.51 log and −1.20 log, respectively) and content (−1.50 log, −1.83 log and −1.42 log, respectively).	[33]
ASEO	4% and 8%	Coated	Chicken nuggets	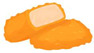	Reduced total viable counts (approximately −2.00 log) and psychrotrophic bacteria (approximately −1.00 log).	[9]
ASEO	0.5%, 1%, and 2%	Film packaging	Chicken breast fillet	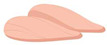	Reduced total viable count, *Staphylococcus aureus* and psychrotrophic bacteria (−1.80 log, approximately −1.50 log, approximately, −1.00 log, respectively).	[57]
ASEO	100 mg/mL	Immersion	Egg	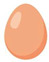	Reduced the load of aerobic mesophilic bacteria, Enterobacteriaceae and moulds and yeasts in the shell (−1.18 log, −1.27 log and −1.34 log, respectively) and content (−1.18 log, −1.27 log and −1.34 log, respectively).	[40]

## Data Availability

Not applicable.
